# Addressing Persistent Logging Hazards Through Accessible Yarding and Loading Safety Training

**DOI:** 10.1007/s44392-026-00077-w

**Published:** 2026-02-24

**Authors:** David A. Hurtado, Jacqueline Boyd, Rachel Madjlesi, Francisca Marrs Belart, Jeffrey Wimer

**Affiliations:** 1 Oregon Institute of Occupational Health Sciences, Oregon Health & Science University, Portland, OR USA; 2 OHSU-PSU School of Public Health, Portland, OR USA; 3 Forestry and Natural Resources Extension Service, Oregon State University, Corvallis, OR USA; 4 Wimer Safety Consulting Inc, Albany, OR USA

**Keywords:** Yarding and loading, Online training, Workforce development

## Abstract

Logging remains one of the most hazardous industries in the United States, with persistent risks associated with yarding and loading operations. In Oregon, these activities account for a substantial proportion of serious and fatal logging injuries, underscoring the need for accessible, foundational safety training for early-career workers. This Forum article describes the development and implementation of the Oregon Yarding and Loading Safety Training, a free, bilingual, self-paced online resource designed to supplement employer-provided instruction. Adapted from the Oregon OSHA Yarding and Loading Handbook, the training translates established technical guidance into an accessible, mobile-friendly format. We outline the rationale for the training, its content and structure, and its relevance to workforce development in Oregon’s logging sector, with implications for broader application in forest operations safety training.

## Introduction

Logging continues to be among the most hazardous industries in the United States, with consistently elevated rates of fatal and nonfatal injuries. Nationally, 249 logging, forest, and conservation workers died from job-related injuries between 2018 and 2022 (Bureau of Labor Statistics 2024). The logging industry is a cornerstone of Oregon’s forest sector and rural economy. Oregon has led the nation in softwood lumber production for over three decades, and the forest sector contributes approximately $20 billion annually to the economy, supporting more than 61,000 jobs across harvesting, transportation, and wood products manufacturing (Johnson 2022; Oregon Forest Resources Institute (OFRI) 2023). Logging is especially vital in rural counties where few comparable industries exist, making workforce stability and safety essential not only for individual workers but also for the resilience of Oregon’s broader forest products supply chain. In Oregon, where steep terrain, cable yarding systems, and complex harvest operations are prevalent, the sector faces recurring fatal incidents that underscore the inherent dangers of falling, yarding, and loading timber.

In 2022, the work-related fatality rate for loggers was 100.7 per 100,000 full-time equivalent (FTE), 27 times the rate for all occupations (3.7 per 100,000 FTE) (Garland et al. 2024). Data from the Oregon Fatality Assessment and Control Evaluation Program (OR-FACE) indicate that from 2003 to 2023, there were 115 logging fatalities. Of those 115 fatalities, 50 (43%) were struck-by incidents including contact with equipment or falling objects such as trees and logs (Oregon Fatality Assessment and Control Evaluation Program (OR-FACE) n.d). Another 40 (35%) involved motor vehicle accidents occurring on both roadways and non-roadways (e.g., logging roads). This category includes motor-vehicle accidents involving logging equipment and pedestrian-related incidents, such as workers being struck and run over. These findings highlight the inherently hazardous nature of logging operations, where steep terrain, remote work locations, variable weather, and heavy machinery can significantly increase risk. They also underscore the continued importance of safety training, effective communication, and hazard assessments to prevent severe and fatal incidents in this industry.

These events illustrate the need for high-quality, accessible introductory training for new workers with limited experience and familiarity with yarding systems. Although employers typically provide on-the-job training, mentorship, and supervision, many new workers enter the field without the structured foundation needed to recognize hazards and understand the principles that govern safe rigging operations. To help address this gap, the OR-FACE Program released the Oregon Yarding and Loading Safety Training (Madjlesi et al. 2025), a free, bilingual, self-paced online curriculum designed to reinforce employer-led instruction and support early-career loggers in acquiring essential safety knowledge (Oregon Occupational Safety and Health Administration n.d.). Although the OR-FACE team developed the training modules and delivery system, the substantive safety content originated from the Oregon OSHA Yarding and Loading Handbook. The goal of the project was not to create new technical guidance, but rather to present established standards in a more accessible, mobile-friendly, and pedagogically structured format for early-career loggers. This Forum Article describes the rationale for the training, its development process, and its implementation through an online learning management platform. The Oregon Yarding and Loading Safety Training is offered free of charge and is publicly accessible through the OR-FACE learning platform at: https://www.supportiveworkplaces.org/oregonyardingandloadingsafetytraining.

## Background and Rationale

The Oregon OSHA Yarding and Loading Handbook has served as a key technical reference for the industry, but many new workers lack the foundational knowledge required to translate its dense regulatory content into practical, field-ready practices. The rapid pace of logging operations can limit opportunities for structured training, particularly in small firms that lack dedicated safety trainers. These realities underscore the need for an introductory resource that is accurate, easily accessible, and designed to be used alongside employer-provided instruction rather than as a replacement.

The training was developed by the OR-FACE Program at the Oregon Institute of Occupational Health Sciences, part of Oregon Health & Science University. Its content was adapted from Chapters 6 and 7 of the Oregon OSHA Yarding and Loading Handbook to ensure alignment with established regulatory practices and terminology. These chapters were selected for adaptation because they introduce core terminology and foundational concepts for workers new to yarding and rigging environments, and because they focus on activities most strongly associated with serious and fatal injuries in logging operations. Prioritizing these chapters allowed the training to address both essential knowledge acquisition and high-risk tasks central to injury prevention.

From an instructional design perspective, the training was developed using principles of adult learning and micro-learning. Rather than relying on lengthy, text-heavy modules, the training was structured around short learning units designed to be completed in brief, focused intervals. Each module articulates clear learning objectives and incorporates interactive elements that prompt learners to reflect on key concepts. Field-based examples help workers visualize how hazards arise and how proper positioning and communication can mitigate risks. The modules were also designed to function on mobile devices, allowing workers to access content before a shift, during breaks, or in remote locations with limited time and connectivity.

Technical oversight was provided by two experienced logging safety educators: Jeff Wimer, Logging Safety Consultant and retired Senior Instructor at Oregon State University, and Francisca Marrs Belart, PhD, Associate Professor and Timber Harvesting Extension Specialist at Oregon State University. Their expertise ensured that the training accurately reflected contemporary logging practices and reinforced essential competencies for rigging crews.

## Training Content

The Oregon Yarding and Loading Safety Training introduces workers to foundational concepts essential to safe yarding and loading operations. The opening module introduces the major types of yarding systems used in Oregon, including high-lead, standing skyline, slack-line, and shotgun configurations. These modules describe the mechanical differences among systems, their functional purposes, and the typical hazard scenarios that accompany them. This helps new workers develop a vocabulary for understanding rigging components and system behavior.

A series of modules on rigging procedures provides detailed explanations of chokers, carriage systems, blocks, and tension management. Practical examples illustrate how correct rigging techniques support safe yarding operations and how improper setup can create hazardous conditions. The module on elevated supports similarly explains the role of tail trees, guy lines, and intermediate supports, with attention to inspection and maintenance practices that reduce the risk of catastrophic system failure.

The next set of modules describes crew roles and responsibilities, helping workers understand how choker setters, rigging slingers, yarder engineers, and landing personnel coordinate their work. By clarifying communication protocols and emphasizing the interdependence of these roles, the training reinforces the importance of shared situational awareness.

Subsequent modules focus on specific common logging hazards, providing context for the varied environmental and operational risks that rigging crews encounter. While these hazards are routinely encountered in logging operations, they remain critically important because they have the potential to cause severe, life-altering, or fatal injuries if not properly recognized and controlled. They emphasize situational awareness, line-of-fire principles, and environmental considerations, including terrain, weather, vegetation, and equipment movement.

## Implementation and Accessibility

The training is hosted on the OR-FACE learning management system and is available to workers and employers at no cost. Users create a basic account and can then complete modules at their own pace. Because the training is delivered online, it requires no specialized equipment and can be accessed on smartphones, tablets, or computers. The training is available both in English and Spanish. Employers may assign the modules to new hires as part of the onboarding process, incorporate them into routine safety meetings, or use specific modules to reinforce safety topics during tailgate trainings. This flexibility supports implementation in a variety of operational settings, including remote field locations and resource-limited environments where formal training infrastructure may be limited.

## Relevance to Forestry and Logging Workforce Development

Oregon’s logging workforce is undergoing demographic changes, marked by the retirement of experienced loggers, the entry of new workers with limited exposure to yarding systems, and an increasing linguistic diversity. These trends challenge traditional mentorship models and underscore the need for standardized foundational training that can be delivered consistently across worksites, while complementing, rather than replacing, mentorship as the core mechanism for transferring experiential safety knowledge in logging operations. The Oregon Yarding and Loading Safety Training offers a cost-effective, accessible way to address these needs by introducing new workers to critical safety concepts before they encounter high-risk situations in the field. Because the training is bilingual and self-paced, it can support a broader segment of the workforce and help reinforce employer expectations. It also has potential applications in forestry and logging education pipelines, including community-college harvesting programs and career-technical education pathways.

Although the training does not confer a formal, industry-recognized credential, participants receive a certificate of completion upon completion of the modules. Associated Oregon Loggers (AOL) currently recognizes this certificate and offers continuing education credit upon receipt of the documentation. In addition, a generic certificate of completion is provided so that participants may submit proof of training to regional or organizational entities that independently determine eligibility for continuing education or other training credit. This approach was intended to support flexibility across jurisdictions while maintaining the training’s primary role as a foundational, supplemental safety resource.

## Conclusion

Cable logging and loading operations remain among the most hazardous components of timber harvesting in Oregon. The combination of steep terrain, high-tension systems, and the need for precise coordination among workers creates an environment where inexperience can have severe consequences. Foundational training is therefore essential for workers entering these settings, particularly as the logging workforce continues to evolve and traditional mentorship structures become more difficult to sustain. The Oregon Yarding and Loading Safety Training offers a practical solution with an accessible, user-friendly, bilingual online format. It reinforces employer instructions, supports early-career workers, and aligns with regulatory guidance while remaining freely available to the industry. As the training is disseminated more broadly and evaluation findings become available, it may serve as a model for similar initiatives in other regions that rely on cable logging systems.
